# The Role of β-Lactam Antibiotics in Treating *Mycobacterium abscessus*: From Laboratory Insights to Clinical Applications and the Case for Clinical Trials

**DOI:** 10.1093/cid/ciaf547

**Published:** 2025-09-30

**Authors:** Khalid M Dousa, Eunjeong Shin, Sebastian G Kurz, Eric J Rubin, Steven M Holland, Kenneth N Olivier, Charles L Daley, Barry N Kreiswirth, Paul S Pottinger, Robert A Bonomo

**Affiliations:** Louis Stokes Cleveland VA Medical Center, Case Western Reserve University, Cleveland, Ohio, USA; Department of Medicine, Case Western Reserve University School of Medicine, Cleveland, Ohio, USA; Louis Stokes Cleveland VA Medical Center, Case Western Reserve University, Cleveland, Ohio, USA; Department of Medicine, Case Western Reserve University School of Medicine, Cleveland, Ohio, USA; Division of Pulmonary, Critical Care and Sleep Medicine, Yale School of Medicine, New Haven, Connecticut, USA; Department of Immunology and Infectious Diseases, Harvard T.H. Chan School of Public Health, Boston, Massachussetts, USA; Laboratory of Clinical Immunology and Microbiology, National Institute of Allergy and Infectious Diseases, National Institutes of Health, Bethesda, Maryland, USA; Division of Pulmonary Diseases and Critical Care Medicine, Department of Medicine, University of North Carolina, Chapel Hill, North Carolina, USA; Division of Mycobacterial and Respiratory Infections, National Jewish Health, Denver, Colorado, USA; Center for Discovery and Innovation, Hackensack Meridian Health, Nutley, New Jersey, USA; Department of Medicine, Division of Allergy & Infectious Diseases, University of Washington School of Medicine, Seattle, Washington, USA; Louis Stokes Cleveland VA Medical Center, Case Western Reserve University, Cleveland, Ohio, USA; Department of Medicine, Case Western Reserve University School of Medicine, Cleveland, Ohio, USA; CWRU-Cleveland VAMC Center for Antimicrobial Resistance and Epidemiology (Case VA CARES), Cleveland, Ohio, USA; Department of Pharmacology, Case Western Reserve University School of Medicine, Cleveland, Ohio, USA; Department of Biochemistry, Case Western Reserve University School of Medicine, Cleveland, Ohio, USA; Department of Proteomics and Bioinformatics, Case Western Reserve University School of Medicine, Cleveland, Ohio, USA; GRECC, Louis Stokes Cleveland Department of Veterans Affairs Medical Center, Cleveland, Ohio, USA

**Keywords:** *Mycobacterium abscessus*, nontuberculous mycobacteria (NTM), β-lactam antibiotics, β-lactamase inhibitors, novel β-lactam/β-lactamase inhibitor combinations

## Abstract

*Mycobacterium abscessus* (*Mab*) is a highly drug-resistant non-tuberculous mycobacterium that presents major treatment challenges, particularly in individuals with structural lung disease. Although historically considered ineffective, β-lactam antibiotics have gained renewed attention due to advances in β-lactamase inhibition and cell wall biology. This review synthesizes more than a decade of work, including in vitro susceptibility studies, biochemical characterization of *Mab*'s β-lactamase (Bla_Mab_) and peptidoglycans synthesis, and published clinical cases supporting the potential role of β-lactam-based regimens. We detail the enzymatic pathways involved in peptidoglycan cross-linking and the dual inhibition of D,D- and L,D-transpeptidases by select β-lactams, as well as the functional impact of inhibiting Bla_Mab_. Novel β-lactamase inhibitors such as durlobactam may further enhance β-lactam efficacy. By integrating laboratory insights with clinical experience, this review provides a comprehensive perspective and informs ongoing efforts to design clinical trials repurposing β-lactam/β-lactamase inhibitor combinations.

## Teaser

This review highlights the evolving role of β-lactam and β-lactamase inhibitor combinations in *Mycobacterium abscessus* therapy, synthesizing preclinical, mechanistic, and emerging clinical data to outline therapeutic promise, challenges, and future research directions.

*Mycobacterium abscessus* (*Mab*), a major nontuberculous mycobacterial (NTM) lung pathogen, includes 3 subspecies: *abscessus*, *massiliense*, and *bolletii*. *Mab* causes colonization or severe lung infections, particularly in CF and bronchiectasis patients. *Mab* is environmentally derived, intrinsically drug-resistant, exhibiting variable susceptibility across subspecies to macrolides, aminoglycosides, quinolones, and tigecyclines [1–5], and cases of human-to-human transmission was also documented [6]. Treatment is challenging due to inducible macrolide resistance (via *erm*[41] in *abscessus* and *bolletii*), requiring prolonged multidrug regimens*. M. massiliense* lacks this gene, making macrolides more effective [7]. Alternatives for resistant strains include aminoglycosides, tigecycline, linezolid, and β-lactams, though efficacy is limited by toxicity and IV delivery [8].

β-Lactams are widely used for bacterial infections but considered ineffective against mycobacteria due to β-lactamase-mediated resistance [9–11]. β-lactamase resistance was first demonstrated for *Mtb*, which has a principal β-lactamase, BlaC [12] that can be inhibited by clavulanate [13]. Subsequently, the β-lactamase of *Mab*, Bla_Mab_, was identified [9], which is poorly inhibited by clavulanate but effectively targeted by newer Diazabicyclooctane (DBO) inhibitors [14]. Current treatment recommendations include imipenem or cefoxitin; however, recent investigations found that alternative β-lactam combinations, with or without Bla_Mab_ inhibition, are highly active [10, 15].

**Figure 1.**
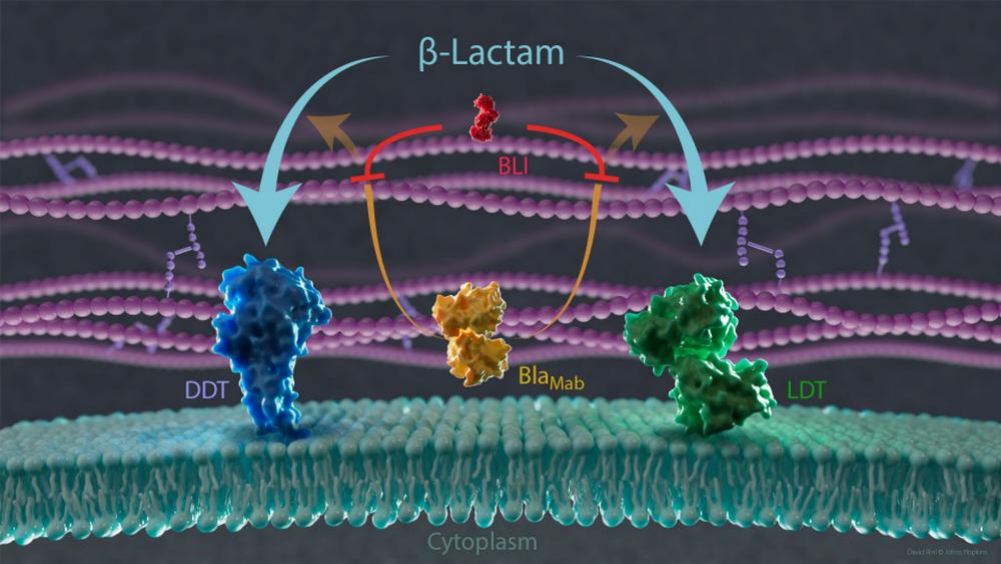
β-lactam targets in peptidoglycan cross-linking: inhibition of D,D-transpeptidases (DDTs) and L,D-transpeptidases (LDTs), β-lactamase inhibitors (BLIs) inhibit β-lactamase enzymes and protect β-lactam antibiotics from degradation. β-lactams target PBPs (also known as DDTs) and LDTs, essential enzymes PG synthesis [21]. The building block of PG in *Mab* is a disaccharide composed of N-acetylglucosamine (GlcNAc) and N-acetylmuramic acid (MurNAc), linked to a stem peptide consisting of L-alanyl-D-glutaminyl-meso-diaminopimelyl-D-alanyl-D-alanine [19]. PG synthesis involves polymerization of disaccharides by transglycosylases and cross-linking of stem peptides by transpeptidases, resulting in a 3-dimensional macromolecular structure. DDTs catalyze the final step of PG synthesis by linking the fourth amino acid of one stem peptide to the third amino acid of an adjacent stem peptide, forming a 4→3 peptide cross-link. However, in mycobacteria, the majority of stem peptides are cross-linked via non-canonical 3→3 linkages, which are generated by LDT enzymes [19, 22, 23]. Given the predominance of LDT-mediated linkages in *Mab*, both enzyme classes are critical targets for β-lactam activity.

In most bacteria, the cell wall comprises glycan strands cross-linked by short peptides ending in D-Ala-D-Ala, a motif mimicked by β-lactams to inhibit penicillin-binding proteins (PBPs). Mycobacteria, however, have a more complex structure, featuring a lipid-rich outer layer anchored to arabinogalactan overlying a Gram-positive-like peptidoglycan (PG) mesh [16]. In addition to classical D,D-transpeptidase (DDT)-mediated cross-linking, *Mab* predominantly utilizes L,D-transpeptidases (LDTs) for PG cross-linking [17–19]. These enzymes present additional β-lactam targets, supporting the rationale for a combination of different β-lactams (Figure 1) [20].

This review outlines the mechanistic basis and clinical rationale for β-lactam therapy, supported by a comprehensive analysis of case reports demonstrating clinical success. We discuss foundational insights, real-world applicability, current limitations, and key challenges and propose directions for future trials to guide clinical practice and research.

## LABORATORY INSIGHTS: Β-LACTAM ACTIVITY AGAINST MYCOBACTERIA, INCLUDING *MAB*

### Mechanisms of Action of β-Lactams

LDTs and PBPs are summarized in Table 1. The 5 LDTs share low sequence identity (9%–25%) [20], suggesting structural and functional differences, including varied β-lactam binding. β-lactam affinities to LDTs and PBPs differ by drug class, affecting antibacterial activity and potential synergy. Although some binding data are available [17, 30–34], the primary targets and roles of LDTs, PBPs, and D,D-carboxypeptidase (DDC)—which converts PG pentapeptides to tetrapeptides to support LDT function— requires further study.

**Table 1.** LDTs, PBPs, and β-Lactam Binding in Mab

Category	Details	Comment
*L,D-transpeptidases (LDTs)*	Five identified: LDT1–LDT5 [24, 25]	High-affinity carbapenem/thiopenem binding [15, 20, 26] High-affinity cephalosporins [15, 20, 26] No low-affinity penicillins (eg, amoxicillin) [26–28]
*Penicillin-binding Proteins (PBPs)*	Eight identified: [29] PonA1 (*MAB*_4901c) PonA2 (*MAB*_0408c) PBPA (*MAB*_0035c) PBPB (*MAB*_2000) PBP-lipo (*MAB*_3167c) *MAB*_0519 DacB1 (*MAB*_3681) DacB2 (*MAB*_3234)	High-affinity carbapenem/thiopenem binding [15, 20, 26] No low-affinity cephalosporins [15, 20, 26] High-affinity penicillins (eg, amoxicillin) [26–28]

### Barrier of β-Lactam Activity in *Mab*

*Mab* has a thick, multilayered cell wall composed of mycolic acids, lipoproteins, glycopeptidolipids, and peptidoglycan-arabinogalactan (Table 2) [35], forming a major barrier to antibiotic entry. Although MspA-like porins facilitate β-lactam diffusion, their low abundance and permeability [2, 30, 39], combined with the waxy outer membrane, significantly limit β-lactam uptake and reduce drug efficacy. Additionally, *Mab* expresses the chromosomal β-lactamase Bla_Mab_ [9], and *M. massiliense* additionally encodes Bla_MMas_ [40]. Bla_Mab_ hydrolyzes most β-lactams, including imipenem, though with variable efficiency [20, 27]. Despite imipenem's low substrate activity [20], increased Bla_Mab_ expression [31] confers resistance, emphasizing the need for β-lactamase inhibitors (BLIs) to maintain efficacy [10]. The potent β-lactamase activity, exceeding that of *Mtb* [35], supports the inclusion of BLIs in β-lactam regimens. β-lactams that penetrate *Mab* may be expelled by efflux pumps [32–34]. While their role in resistance to other antibiotics is established [41–43], their contribution to β-lactam resistance appears minimal but remains poorly understood, warranting further study (Table 2).

**Table 2.** Barriers to β-Lactam Therapy in *Mab*

Barrier	Mechanism/Details	Impact on β-lactam Therapy
Thick, complex cell wall	Composed of fatty acids, mycolic acids, lipoproteins, glycopeptidolipids, peptidoglycan (PG), and arabinogalactan	Physically impedes β-lactam entry due to its hydrophobic and multilayered nature
Limited porin-mediated permeability	MspA-like porins are scarce and have low permeability	Restricts diffusion of hydrophilic β-lactams into the bacterial cell
β-lactamase activity	Bla_Mab_ (ubiquitous), Bla_MMas_ (in *Mab* subspecies *massiliense*)	Hydrolyze β-lactams, including imipenem; contributes significantly to resistance
Overexpression of β-lactamases	Increased Bla_Mab_ expression in later isolates	Correlates with rising resistance even to poor Bla_Mab_ substrates like imipenem
Efflux pumps	Includes TetV-like, *MAB*_2355c, *MAB*_1409c, MAB_1846, MmpS/L, *MAB*_4384 [34, 36–38]	Role in β-lactam resistance remains unclear; implicated more in resistance to other drug classes
Lack of effective β-lactamase inhibitors (BLIs)	Most current β-lactam therapies combinations lack co-administered effective BLIs	Limits the ability of β-lactams to remain active in the presence of potent β-lactamases

### *In Vitro* Studies

*In vitro* studies have assessed the efficacy of β-lactams against *Mab* using various approaches, including minimum inhibitory concentration (MIC) testing, checkerboard assay, time-kill assays, and the hollow fiber infection model (HFIM). These studies have demonstrated that single β-lactam exhibit limited activity against *Mab*, and among β-lactams, imipenem has shown the highest activity. This limited activity is largely attributed to the presence of β-lactamases, Bla_Mab_, which efficiently hydrolyze β-lactams, thereby diminishing their antibacterial effect [20, 27]. However, new insights suggest that the addition ofx a second β-lactam (dual β-lactam; DBL) or a BLI can enhance β-lactam efficacy against *Mab* [10, 14, 15, 20, 27, 28, 44–50]. MIC testing is a simple and scalable method for assessing β-lactam activity and synergy but does not capture dynamic killing or regrowth. Time-kill assays address this by tracking viability over time, highlighting regrowth with monotherapy and sustained killing with combinations like imipenem–avibactam or –durlobactam [12, 17, 51]. The HFIM better simulates human PK and reveals improved efficacy with novel BL/BLI regimens, yet, remains underused in *Mab* research because of drugs instability. Broader HFIM use is essential for optimizing dosing and reducing resistance [52, 53]. Recent investigations have addressed parameters to be followed to replace labile β-lactams antibiotics [41].

#### Synergistic Effects of DBL

**Figure 2.**
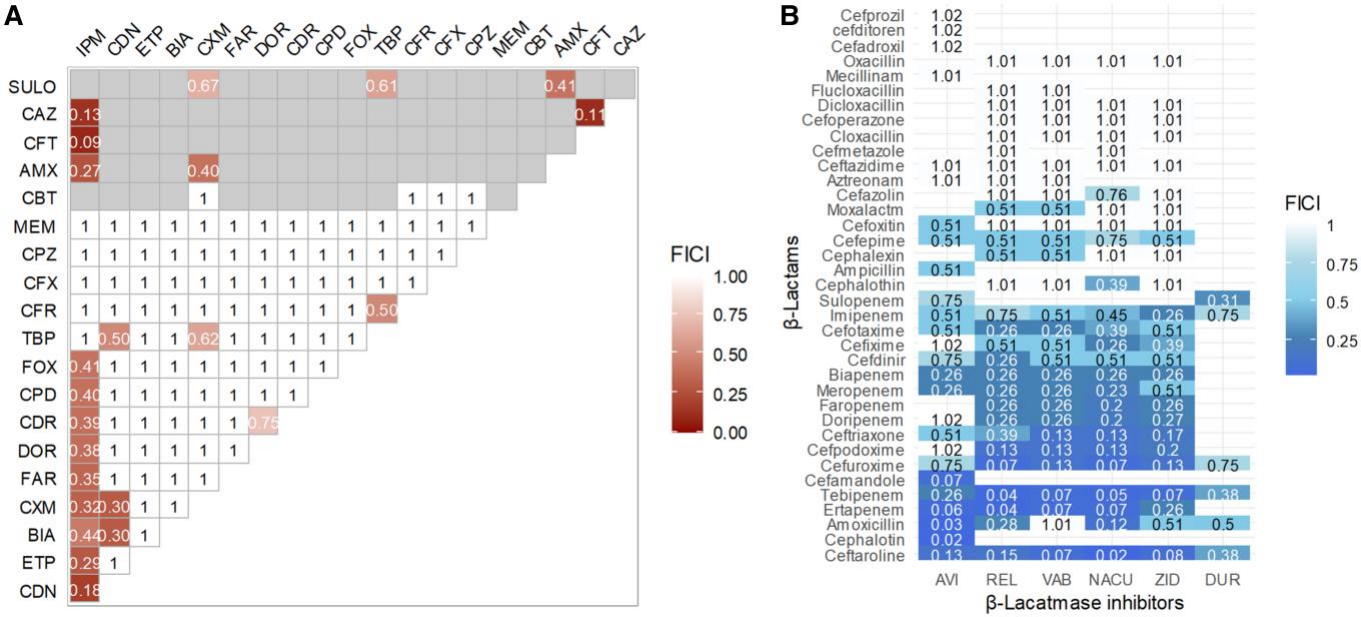
Summary of in vitro synergy of DBL (*A*) and BL/BLI (*B*). The fractional inhibitory concentration index (FICI) values for each combination are presented, with lower values depicted in darker red to indicate stronger synergy. A FICI value of ≤0.5 denotes synergy, values between 0.5 and <1 indicate an additive effect, and values of ≥1 represent indifference. AMX (amoxicillin), AVI (avibactam), BIA (biapenem), CAZ (ceftazidime), CBT (ceftibuten), CDN (cefditoren), CDR (cefdinir), CFR (cefadroxil), CFT (ceftaroline), CFX (cefixime), CPD (cefpodoxime), CPZ (cefoperazone), CXM (cefuroxime), DOR (doripenem), DUR (durlobactam), ETP (ertapenem), FAR (faropenem), FOX (cefoxitin), IMP (imipenem), MEM (meropenem), NACU (nacubactam), REL (relebactam), TBP (tebipenem), VAB (vaborbactam), ZID (zidebactam).

DBL therapy has shown promise against Mab, with ∼10 studies evaluating combinations—mainly carbapenems with cephalosporins, penems, or penicillins. Among 117 tested pairs (Figure 2*A*) [10, 15, 20, 27, 28, 44–46], 23 showed synergy, with imipenem involved in nearly half. Synergy likely arises from targeting multiple cell wall enzymes (LDTs and PBPs) [20]. This target redundancy, as proposed by Dousa et al [15] and supported by Shin et al [10] may enhance bacterial killing and binding affinity in DBL regimens.

#### Synergistic Effects With BLIs

Bla_Mab_ shows strong β-lactamase activity, and traditional BLIs like clavulanic acid, tazobactam, and sulbactam are largely ineffective—clavulanic acid may even be antagonistic [14, 35]. In contrast, DBO inhibitors (eg, avibactam, durlobactam [30, 33]) inactivate Bla_Mab_ and enhance β-lactam activity (Figure 2*B*) [12, 16, 17, 28, 30, 54–57]. Avibactam and durlobactam also bind to LDTs and PBPs [10], potentially contributing β-lactam-like effects. These findings provide a strong biochemical rationale for their promise in future β-lactam-based therapies [12, 17].

### Key Challenges in *In Vitro* Studies

Many β-lactams are unstable under laboratory conditions [41, 46], degrading during the extended incubation times required for *Mab* assays, which may lead to overestimated MICs and underestimated potency. Static in vitro assays (eg, MIC, checkerboard, time-kill) fail to replicate the dynamic PK/PD of β-lactams in vivo. While HFIM offers a more physiologic model, its use is limited by high costs, media requirements, and the need for frequent drug replenishment. Variability in assay design and the disconnection between lab conditions and in vivo environments—such as biofilms or intracellular niches—further complicate efficacy assessment. These challenges emphasize the need for improved, translational models that account for drug stability, PK/PD dynamics, and host-related factors.

### Preclinical and Animal Models

Various animal models, including zebrafish, Drosophila, and mice, have been used to study *Mab* infections [23–26, 29, 36–38, 56, 58], though most cannot fully to fully replicate human pulmonary disease. Murine models often emphasize non–β-lactam antibiotics, with limited data on β-lactam combinations. An immunosuppressed aerosol-infection mouse model better mimics human lung infection [59] and has been used to test BL/BLI and DBL regimens. However, PK/PD studies remain limited. Given the altered drug penetration in diseased lungs and short β-lactam half-lives, infection-specific PK/PD studies are critical but challenging, especially in chronic or immunocompromised models. Animal models are vital for studying drug distribution to target tissues, which cannot be directly assessed in humans. However, lung pathology in mouse and humans with *Mab* infections may alter drug penetration, making infection-specific PK/PD studies essential. Challenges include the short half-life of β-lactams, requiring frequent dosing to maintain adequate time above the MIC (%T > MIC), and the difficulty of sustaining long-term exposure in chronic model. Short treatment durations and the high bacterial clearance in immunocompetent mice can underestimate efficacy, while immunosuppressed models, though more representative, must be carefully managed to avoid excessive morbidity.

### CLINICAL EVIDENCE: Β-LACTAM USE IN MAB INFECTIONS AND DRUG SUSCEPTIBILITY TESTING

Management of *Mab* infections—both pulmonary and extra-pulmonary—remains highly challenging, requiring prolonged, toxic multidrug regimens complicated by DDI and limited clinical evidence-especially in vulnerable populations such as the immunocompromised, pregnant, and pediatric patients. Clinicians frequently find themselves navigating a landscape of limited evidence, therapeutic uncertainty, and high-stakes decision-making. Alarmingly, treatment outcomes for drug-susceptible *Mab* subsp. *abscessus* are often worse than those for multidrug-resistant tuberculosis (MDR-TB) and may approximate those of extensively drug-resistant TB (XDR-TB) [60]. In cases of clinical deterioration, including progression to mycobacteremia, patients often decline rapidly and require urgent, decisive intervention. The current American Thoracic Society (ATS) and Infectious Diseases Society of America (IDSA) guidelines provide some direction on managing drug-resistant *Mab* infections, leaving clinicians with little consensus and driving them to explore individualized, and at times experimental, therapeutic strategies.

In recent years, the use of dual β-lactam therapy—with or without the addition of BLIs—has emerged as an area of growing interest. Encouraged by promising in vitro data and the well-established safety, PK/PD, and tolerability profile of β-lactams, clinicians have increasingly turned to select study centers laboratories for synergy testing to guide dual β-lactam regimens.

While formal clinical trials remain lacking, accumulating evidence from case reports and case series suggests that DBL therapy with or without BLIs used as a part of multi-drug regimen may be both effective and well tolerated. Clinical reports have described successful eradication of *Mab* in cases previously considered refractory to treatment. Table 3 provides a summary of the published clinical cases below employing this strategy. The growing interest in this approach is largely fueled by the paucity of viable alternatives, combined with the high relapse and failure rates associated with conventional therapy.

**Table 3.** Reported Cases of β-Lactam Use With Clinical Success in the Treatment of *Mab* Infections

Publication	Site of Infection	*M. Abscessus* Subspecies	Macrolide Resistance	Antimicrobial Regimen	β-Lactam/β-Lactamase Inhibitor	Adverse Side Effects	Comment
Daley, Charles 2022	Pulmonary, cavitary disease	*abscessus*	Yes	Amikacin, clofazimine	Imipenem, ceftaroline	None	Unpublished data, oral abstract presentation in Infectious Diseases Week 2022 History of cavitary pulmonary tuberculosis post segmentectomies
Alahmadi et al 2023 [58]	Pulmonary	*massiliense*	Yes *Rrl* gene mutation	Amikacin, clofazimine, omadacycline, tedizolid	Imipenem, ceftaroline	Elevated liver enzymes	Imipenem and ceftaroline used for 2 m
Wolf et al 2023 [61]	Central nervous system (meningitis)	*abscessus*	No	Azithromycin, eravacycline, tedizolid	Imipenem, ceftaroline	None	Immunocompetent, Infection developed after intrathecal injection of donor umbilical cord stem cells programmed to treat multiple sclerosis in Baja California, Mexico
Alhebsi et al 2024	Skin and soft tissue infection	*abscessus*	No	None	(Imipenem-ceftaroline) (amoxicillin-cefdinir)	None	Unpublished data, oral abstract presentation in ESCMID 2024. Abdominal wall infection in a pregnant women cured with only dual β-lactams
Kalyatanda et al 2024 [62]	Mixed	*abscessus, massiliense* and *bolletii*	Mixed	Several	(Imipenem-amoxicillin) (Imipenem-cefdinir), (Imipenem-cefuroxime), (Imipenem-relebactam with amoxicillin) (Meropenem and cefoxitin)	Gastrointestinal side effects, elevated liver enzymes, and eosinophilia	Results in 19 patients, severely immunocompromised and some patients with organ transplants Therapy guided by drug susceptibility testing
Becken et al 2024 [63]	Pulmonary	*abscessus*	No	Linezolid, bedaquiline, eravacycline, clarithromycin	(Meropenem-ceftaroline) (Imipenem-cefdinir)	None	Pulmonary infection in a child. Complete clinical, microbiological, and radiographic cure in 8 m total
Moguillansky et al 2023 [64]	Pulmonary	*massiliense*	No	Clofazimine, azithromycin, liposomal amikacin	Imipenem-relebactam-amoxicillin	None	Imipenem and amoxicillin were discontinued after 2 m, AFB culture conversion in 9 m. Therapy completed 12 months after culture conversion
Vogiatzoglou et al 2024 [65]	Pulmonary, cavitary disease	*massiliense abscessus*	No	Amikacin, tigecycline, azithromycin, linezolid, clofazimine	Imipenem-relebactam- amoxicillin	None	Two cases, cavitary and nodular bronchiectatic pulmonary infection Clinical and radiographic improvement noted after 3 m of treatment
Cristinziano et al 2024 [66]	Skin and soft tissue infection	*abscessus*	Yes	Eravacycline, linezolid, tedizolid, clofazimine, omadacycline, amikacin, bedaquiline	Meropenem-ceftazidime avibactam	None	Lung transplant recipient Treated with 2 phages, including one with epigenetic modifications
Shirley et al 2025 [67]	Peritonitis	*abscessus*	No	Amikacin, linezolid, azithromycin	Meropenem-ceftaroline, amoxicillin, and cefdinir	None	A case report of peritoneal dialysis-associated peritonitis caused by *Mycobacterium abscessus* in a child, successfully treated with dual β-lactam combination therapy
Shimamura et al 2025 [68]	Mixed	*abscessus*	Yes	Tigecycline, amikacin, linezolid, doxycycline, eravacycline, clofazimine, bedaquiline	Meropenem and ceftaroline, meropenem and amoxicillin.	…	Successful treatment of macrolide-resistant *Mycobacterium abscessus* infection using multi-drug regimens including dual β-lactams and phage therapy: case reports in 2 children

The clinical application in the reported cases of dual β-lactam therapy in *Mab* infections is inherently complex due to the heterogeneity in drug selection, dosing strategies, and treatment durations. In the absence of standardized protocols or large-scale clinical trials, combinations are often guided by in vitro synergy testing; however, even when such data are available, the final antimicrobial regimen is frequently determined by the treating physician's clinical judgment. Practical considerations—including drug availability, insurance coverage, and cost—play a significant role in therapeutic decision-making. Additionally, patient-specific factors such as drug tolerability, intravenous access, and the feasibility of administering 1 versus 2 intravenous β-lactams often influence regimen selection. This variability underscores the urgent need for prospective studies and consensus guidelines to optimize and standardize the use of dual β-lactam therapy in *Mab* management.

Media selection is also critical, as the use of different broth formulations (eg, Middlebrook 7H9 with or without Tween 80, cation-adjusted Mueller–Hinton broth) can yield variable MICs due to differences in growth rates and drug–bacteria interactions [69]. The absence of standardized drug susceptibility testing (DST) for *Mab* remains a major barrier to patient care, with variability in broth microdilution methods, commercial panels, media choice, inoculum preparation, and incubation conditions leading to inconsistent MICs and difficult interpretation. β-lactam testing is particularly problematic, as media selection strongly influences results: while 7H9 broth is preferred over Mueller–Hinton, the addition of Tween 80 can artificially enhance drug penetration and overestimate activity [42]. *In vitro* MICs of both standard of care antibiotics and β-lactams often fail to predict clinical outcomes, as they do not fully account for host factors, PK/PD, or tissue penetration, further limiting their utility as a standalone guide. Therefore, MICs require contextual interpretation. For imipenem, even high MICs may remain clinically relevant due to time-dependent activity and achievable %T > MIC with optimized dosing. For cefoxitin, MICs are typically higher and activity is often bacteriostatic [43], so values are interpreted comparatively and in the context of PK/PD indices, achievable concentrations, and clinical response rather than as strict cutoffs.

Additional challenges include the lack of harmonized breakpoints (CLSI/EUCAST often misalign with wild-type distributions), drug instability during prolonged incubation, and absence of validated synergy testing despite reliance on combination therapy. Although molecular assays (eg, *erm[41]* or aminoglycoside resistance mutations) offer rapid resistance detection, they cannot substitute for phenotypic testing, given non-mutational mechanisms and incomplete coverage [8]. Together, these gaps highlight the urgent need for harmonized protocols, validated interpretive criteria, and clinician-driven studies—including standardized cohorts and clinical trials—to improve reproducibility, comparability, and ultimately patient outcomes in NTM infections. Moreover, a major diagnostic gap is the limited use of whole-genome sequencing (WGS) to distinguish treatment failure from reinfection. Relapsed isolates are often assumed to be the same strain, but without WGS this cannot be confirmed. Given the ubiquity of these organisms in environmental niches such as piped water, reinfection is a likely but under-recognized possibility.

As with nearly all antimicrobials used for NTM infections, therapy is not strongly supported by randomized controlled trials or robust evidence, and considerable uncertainty remains regarding optimal dosing and treatment duration. This is equally true for β-lactam use in NTM disease—despite representing a newer therapeutic approach. Nonetheless, based on our experience, guided by susceptibility results and with both reported and anecdotal cases, the combinations summarized in Table 4 potentially hold promise and warrant systematic evaluation in laboratory studies and clinical trial settings to better guide therapy. As with standard of care, an intravenous course for at least 2 months if tolerated is preferred, followed by step-down to an oral regimen as outlined in the table. We consider double β-lactam therapy, with or without a β-lactamase inhibitor, as a single component to be incorporated into a multidrug regimen in accordance with current guidelines. When a second β-lactam is added to a penem—the cornerstone of therapy—it is intended to augment and restore the activity of the penem. Until formal, controlled studies are available, this approach remains provisional. Importantly, this strategy may also help minimize the toxic side effects of some agents currently included in guidelines, given the favorable safety profile of β-lactams compared with other drugs.

**Table 4.** Potential β-Lactam Treatment Approaches for NTM: Reported and Clinical Experience

Antimicrobials	Doses	Route	Frequency	Comment
**Imipenem + ceftaroline** [58, 70]	Imipenem 500–100 0 mg Ceftaroline 600 mg	IV IV	Twice daily Twice daily	Difficult to administer as both are IV; generally well tolerated, though liver enzyme elevations observed. Typically given for ∼2 m, then stepped down to oral amoxicillin–cefuroxime to complete therapy
**Imipenem + cefuroxime**	Imipenem 500–1000 mg Cefuroxime 500 or 750 mg	IV Oral	Twice daily Twice daily	Cefuroxime exhibits moderate oral bioavailability
**Imipenem + Cefdinir**	Imipenem 500–1000 mg Cefdinir 300 mg	IV Oral	Twice daily Twice daily	Cefdinir has low oral bioavailability
**Meropenem + amoxicillin** [68]	Meropenem 500 mg-1 Amoxicillin 500 mg or 1 g	IV Oral	Twice daily Three times daily	Amoxicillin is not stable against the Bla_Mab_ β-lactamase produced by *Mab.* Therapy must be guided by susceptibility testing
**Imipenem-relebactam + amoxicillin** [64, 71]	Imipenem-relebactam 1.25 mg (imipenem 500 mg, cilastatin 500 mg, and relebactam 250 mg) Amoxicillin 500 mg or 1 g	IV Oral	Twice daily Three times daily	We observed a consistent and robust inhibitory effect, with nearly all tested isolates demonstrating synergy. However, the high cost of imipenem–relebactam and limitations in patient insurance coverage remain significant hurdles
**Ceftazidime-avibactam + meropenem** [66]	Ceftazidime-avibactam 2.5 g (2 g ceftazidime plus 0.5 g) Meropenem 1–2 g	IV IV	Twice daily Twice daily	Ceftazidime is not active against *Mab,* paired with avibactam to inhibit Bla_Mab_. The high cost of ceftazidime–avibactam, especially with prolonged (1–2 m) use, is an obstacle
**Durlobactam-sulbactam + imipenem** [27]	Durlobactam-sulbactam 2 g (1 g of sulbactam and 1 g of durlobactam) Imipenem 500–1000 mg	IV IV	No clinical experience	Based on robust biochemical analyses, this is the most in vitro active combination against *Mab*. However, clinical experience, and frequency of administration in mycobacterial infections remain undefined. Durlobactam–sulbactam is currently limited to hospital settings due to administration challenges, and high cost remains a major barrier
**Sulopenem-probenecid + cefuroxime** [15]	Sulopenem-probenecid (sulopenem etzadroxil 500 mg with probenecid 500 mg) Cefuroxime 500 or 750 mg	Oral Oral	Twice daily Twice daily	Both agents are stable against Bla_Mab_. No clinical experience. Sulopenem etzadroxil and probenecid is now FDA approved and commercially available in the United States as of October 2024, under the brand name ORLYNVAH, an excellent option for oral step-down therapy, high cost is a major barrier
**Cefuroxime or cefdinir + amoxicillin** [67]	Cefdinir 300 Amoxicillin 500 mg or 1 g Cefuroxime 500 or 750 mg	Oral Oral	Twice daily Three times daily	Minor synergy observed between these oral agents; suitable as step-down therapy or as an initial regimen when IV use is limited, guided by drug susceptibility testing

The general principle is to combine 2 agents that are stable against Bla_Mab_, in which case a β-lactamase inhibitor (BLI) is unnecessary. If one of the agents is unstable (eg, amoxicillin), then a BLI such as relebactam or avibactam is required.

Abbreviations: IV, intravenous; NTM, nontuberculous mycobacteria.

Meropenem may be substituted for imipenem; however, it is less stable against the Bla_Mab_ β-lactamase produced by *Mab*. The evolution of high imipenem resistance in *Mycobacterium abscessus* has been reported with β-lactam therapy; in such cases, the addition of a β-lactamase inhibitor is necessary [31]. Anecdotally, early use of this approach shortened therapy duration and hastened culture conversion [62]. For amoxicillin, 1 g 3 times daily is preferred if tolerated based on pharmacokinetic/pharmacodynamic (PK/PD) data.

## CHALLENGES IN TRANSLATING LAB FINDINGS TO CLINICAL PRACTICE

### Combination Therapy to Enhance β-lactam Efficacy

Given the limited efficacy of β-lactam monotherapy against *Mab*, combination strategies have been explored to overcome resistance and improve bacterial killing. These approaches primarily focus on pairing β-lactams with other antimicrobials or immune-modulating agents to enhance their activity.

#### Combinations With Non-β-Lactam Antibiotics

Macrolides inhibit protein synthesis but show variable efficacy in *Mab* due to inducible resistance [4, 62, 66, 67]. Aminoglycosides disrupt bacterial translation and are often used in combination regimens for *Mab* infections [51, 72]. Though often combined with β-lactams, recent studies show limited synergy [15, 54]. Tigecycline combined with imipenem or cefoxitin has shown promise and warrants further evaluation in animal models [55].

#### For Additional Details on Combinations With Immune-Modulating Agents and Drug–Drug Interactions

Please refer to the Supplementary Material, which includes the associated references.

### Challenge in PK/PD Aspects

Traditional in vitro models, including MIC, checkerboard, and static time-kill assays, rely on fixed drug concentrations that do not reflect the dynamic pharmacokinetics observed in patients, limiting their translational relevance. Although the HFIM allows for dynamic PK/PD modeling and extended exposure studies, it is constrained by high cost, limited accessibility, and technical challenges such as the need for frequent media replacement due to the short half-life of β-lactams. Moreover, all in vitro systems lack immune components, protein binding, and tissue penetration, reducing their predictive value for clinical outcomes. Animal models face additional challenges in modeling *Mab* infection, including difficulty achieving and maintaining clinically relevant lung bacterial burdens (∼10⁷ CFU), variability in infection dynamics, and interspecies differences in drug pharmacokinetics. Most studies are short term, limiting assessment of long-term efficacy and resistance suppression, which are critical for treating chronic *Mab* infections. β-Lactams, although optimized for rapidly growing bacteria, generally exhibit poor penetration into *M. abscessus*–infected tissues and intracellular niches; however, emerging data demonstrate promising macrophage and THP-1 cell penetration [46]. Conventional PK metrics like plasma T > MIC may not predict efficacy in pulmonary infections due to high MICs and frequent sub-therapeutic exposures. High doses may be needed but are limited by toxicity. Additionally, complex combination regimens are challenging to optimize due to differing PK profiles, tissue distribution, and drug–drug interactions (DDIs), making clinical translation of in vitro and in vivo synergy difficult.

## FUTURE DIRECTIONS AND POTENTIAL FOR Β-LACTAM-BASED THERAPIES

DBL and BL/BLI combinations are promising antimycobacterial approaches that offer hope against resistant *Mab*. Current guidelines for *Mab* treatment include cefoxitin or imipenem without a partner BLIs, despite the presence of broad-spectrum Bla_Mab_. Given the unique physiology of *Mab*, standard media may not accurately predict in vivo efficacy. Improving in vitro susceptibility testing to better recapitulate the host environment—alongside clinical trials incorporating diverse media and correlating with clinical outcomes—will enhance treatment strategies. Synergy testing in physiologic media and integrated PK/PD modeling are essential to bridge the gap between in vitro activity and in vivo success, representing a critical yet underexplored research need. Despite the long-standing clinical use of β-lactams and their well-established dosing strategies, in vitro and in vivo data specific to *Mab* remain limited. To effectively translate β-lactam use into clinical success for *Mab* infections, especially pulmonary disease, clinical studies are needed to evaluate their efficacy under infection-specific conditions. This includes investigating drug penetration into infected lung tissue, where host–pathogen interactions and granulomatous architecture may alter drug distribution. Additionally, deeper mechanistic insights into β-lactam activity and resistance pathways are essential to guide rational combination strategies and overcome therapeutic limitations.

Future strategies to enhance β-lactam efficacy against *Mab* will require a multifaceted approach. One promising direction is the development and optimization of BLIs that can more effectively neutralize Bla_Mab_. In many current regimens, BLIs are paired with β-lactams that exhibit minimal, if any, intrinsic activity against *Mab*—for example, avibactam combined with ceftazidime or durlobactam paired with sulbactam—both of which are administered intravenously, adding complexity given the prolonged duration of therapy. An ideal regimen would pair an oral BLI with an oral cephem or penem, thereby simplifying therapy and potentially improving patient adherence. However, no oral BLI currently demonstrates activity against Bla_Mab_, and most penems- that are stable against Bla_Mab_ are limited to intravenous administration. Another approach involves the structure-guided design of novel β-lactams with enhanced affinity for essential LDTs/PBPs and improved resistance to hydrolysis by Bla_Mab_. Additionally, combining β-lactams with adjuvant antimicrobial agents that enhance intracellular penetration or further disrupt cell wall synthesis may bolster therapeutic effectiveness. Advances in nanoparticle drug delivery systems also hold promise by increasing tissue penetration and drug stability, potentially overcoming PK limitations [56].

Designing a clinical trial for β-lactam–based therapy in *Mab* requires careful consideration of multiple factors. Firstly, the target population must be clearly defined—such as adult versus pediatric patients, treatment-naïve versus experienced, or refractory cases—is essential. Inclusion of diverse groups—such as CF versus non-CF bronchiectasis—can affect drug response and limit generalizability, as these subgroups differ PK, comorbidities, and disease presentation. Patients with chronic obstructive pulmonary disease, nodular versus cavitary radiographic patterns, or lobar versus multi-lobar disease may have markedly different prognoses and responses to therapy. Trial design is further complicated by frequent co-infections (eg, *Aspergillus*, MAC), which may confound drug effects. Including MAC cases may dilute *Mab*-specific findings, making it harder to detect signal in subgroup analyses. Another consideration is drug susceptibility, particularly macrolide and amikacin resistance. Resistant cases are important to include but may lower overall success rates. Disease stage also affects outcomes, as patients with early-stage disease may improve clinically, while those with advanced disease may not, despite microbiological response. Thus, outcome measures must be flexible accounting for the heterogeneity of *Mab* disease and treatment response. These may include a combination of microbiological (eg, time to culture conversion, sustained negativity), clinical (eg, symptom improvement, exacerbation frequency, radiographic changes) and patient-reported outcomes (eg, quality of life, functional status). Flexibility is required to capture meaningful benefit across diverse patient subgroups. Secondly, while guideline-based therapy may serve as a comparator, it is important to note that current recommendations are based primarily on limited observational data, lacking validation through randomized controlled trials. Therefore, designing a non-inferiority trial is not feasible. Instead, clinical trials may pursue a superiority design but establishing a meaningful and justifiable superiority margin introduces additional complexity.

Selection of the primary endpoint is also critical. Although culture conversion remains the standard microbiological outcome, its correlation with clinical improvement is debated. Alternative or adjunctive endpoints—such as the desirability of outcome ranking (DOOR) [57], patient-reported outcomes (PROs) [73], or radiographic stability—may offer a more comprehensive assessment in the context of chronic pulmonary disease. Furthermore, the timing of culture assessments (eg, at 3, 6, or 12 months) can substantially influence outcome interpretation. Trial duration presents another challenge: prolonged studies may reduce adherence, increase operational complexity, and exclude patients with the highest need. In monotherapy trials, concerns regarding resistance emergence and confounding by background therapy must also be addressed. Finally, whether the trial is Phase II or Phase III will impact key design elements, including inclusion criteria, endpoint selection, and overall trial scope. In 2019, an FDA workshop convened experts to address challenges in antimicrobial development for NTM [74]. A key recommendation was to reduce study population heterogeneity to improve outcome interpretation. Currently, no clinical trials DBL—with or without a DBO-BLIs—are listed on ClinicalTrials.gov, though one is in development. A concerted effort is urgently needed—including well-designed clinical trials, centralized susceptibility testing, laboratory validation of synergy assays, and systematic reporting of both positive and negative outcomes by clinicians using double β-lactams—to establish a clear and rational therapeutic approach for *Mab* infection.

## CONCLUSION

During the past decade, substantial progress has been made in understanding β-lactam therapy for *Mab*, with pre-clinical synergy data, clinical tolerability, and case reports suggesting their potential. However, clinical translation remains limited by inconsistent susceptibility testing, variable dosing strategies, and a lack of prospective trials. For future development, both agents in DBL or β-lactam/β-lactamase inhibitor combinations should demonstrate intrinsic antimicrobial activity against *Mab*. Optimizing β-lactam use in *Mab* will require physiologically relevant drug susceptibility testing methods, predictive animal model, further drug development, and rigorously designed clinical trials that account for disease complexity. To this epilogue, β-lactams represent a promising yet underutilized strategy in *Mab*, and progress will depend on coordinated efforts across research, clinical, pharmaceutical, and regulatory sectors to drive effective, evidence-based therapies forward.

## Supplementary Material

ciaf547_Supplementary_Data

## Supplementary Data

Supplementary materials are available at *Clinical Infectious Diseases* online. Consisting of data provided by the authors to benefit the reader, the posted materials are not copyedited and are the sole responsibility of the authors, so questions or comments should be addressed to the corresponding author.
