# Supplementary material for: The Role of β-Lactam Antibiotics in Treating Mycobacterium abscessus: From Laboratory Insights to Clinical Applications and the Case for Clinical Trials
Source: Clin Infect Dis. 2025 Sep 30;82(1):e135–45. doi: 10.1093/cid/ciaf547 (PMC13375569; doi:10.1093/cid/ciaf547)
Supplement: ciaf547_Supplementary_Data [file ciaf547_supplementary_data.docx]

**Additional details on combinations with immune-modulating agents and drug–drug interactions (DDIs)**

1. ***Combinations with Immune-Modulating Agents:*** *Mab* has the ability to persist within macrophages, evading host immune clearance(1, 2). Autophagy-inducing agents such as rapamycin and metformin may enhance bacterial clearance by promoting phagolysosomal fusion and intracellular killing(3). The combination of β-lactams with autophagy inducers is an emerging area of interest. A novel resveratrol analogue, V46, has demonstrated potent autophagy activation and antimicrobial activity in macrophage and murine models, enhancing bacterial clearance and synergizing with rifabutin.(4) Additionally, immune checkpoint inhibition, specifically nivolumab, has shown promise in a case report: a patient with *Mab* pulmonary infection experienced clinical improvement after nivolumab administration for lung cancer.(5) While clinical trials in this area are limited, these findings suggest potential benefits of combining β-lactams with host-directed therapies, warranting further preclinical and clinical investigation. Moreover, chronic *Mab* infections may be associated with immune exhaustion, and immune checkpoint blockade has been proposed as a strategy to restore immune function. Combining β-lactams with immune checkpoint inhibitors (PD-1/PD-L1 Inhibitors) may enhance bacterial clearance, though preclinical validation is needed.(6)
2. **Drug-drug interaction (DDI)**

Combination therapy is a cornerstone in the treatment of *Mab* to enhance efficacy, however, the potential for DDI necessitates careful consideration. β-Lactam antibiotics are generally considered safe, and most reported side effects of DBL or BL/BLI combinations, are mild, including gastrointestinal disturbances and headaches. Notably, in a randomized clinical trial by *Jiao Y et al.*, DBL combinations have been associated with a lower incidence of nephrotoxicity compared to β-lactam-aminoglycoside combinations(7). However, since most β-lactams are predominantly eliminated via renal excretion, combining two β-lactams, or β-lactams with tetracyclines—both of which are excreted through the renal anionic transport system—could result in competition for renal clearance.(8) This interaction may lead to increased systemic exposure of β-lactams, potentially enhancing their efficacy but also increasing the risk of renal toxicity. Therefore, monitoring renal function is crucial when using these combinations, particularly in patients with pre-existing renal impairment.

1. Abdelaal HFM, Chan ED, Young L, Baldwin SL, Coler RN. Mycobacterium abscessus: It's Complex. Microorganisms. 2022;10(7).

2. Ferrell KC, Johansen MD, Triccas JA, Counoupas C. Virulence Mechanisms of Mycobacterium abscessus: Current Knowledge and Implications for Vaccine Design. Front Microbiol. 2022;13:842017.

3. Gupta A, Sharma D, Meena J, Pandya S, Sachan M, Kumar S, et al. Preparation and Preclinical Evaluation of Inhalable Particles Containing Rapamycin and Anti-Tuberculosis Agents for Induction of Autophagy. Pharm Res. 2016;33(8):1899-912.

4. Sapkota A, Park EJ, Kim YJ, Heo JB, Nguyen TQ, Heo BE, et al. The autophagy-targeting compound V46 enhances antimicrobial responses to Mycobacteroides abscessus by activating transcription factor EB. Biomed Pharmacother. 2024;179:117313.

5. Ishii S, Tamiya A, Taniguchi Y, Tanaka T, Abe Y, Isa SI, et al. Improvement of Mycobacterium abscessus Pulmonary Disease after Nivolumab Administration in a Patient with Advanced Non-small Cell Lung Cancer. Intern Med. 2018;57(24):3625-9.

6. Li G, Yang H, Ke T, Tan N, Du X, Duan X, et al. Escherichia coli combination with PD-1 blockade synergistically enhances immunotherapy in glioblastoma multiforme by regulating the immune cells. J Transl Med. 2025;23(1):164.

7. Jiao Y, Moya B, Chen MJ, Zavascki AP, Tsai H, Tao X, et al. Comparable Efficacy and Better Safety of Double beta-Lactam Combination Therapy versus beta‑Lactam plus Aminoglycoside in Gram-Negative Bacteria in Randomized, Controlled Trials. Antimicrobial agents and chemotherapy. 2019;63(7).

8. Cattaneo D, Gervasoni C, Corona A. The Issue of Pharmacokinetic-Driven Drug-Drug Interactions of Antibiotics: A Narrative Review. Antibiotics (Basel). 2022;11(10).
